# Improving the performance of floating gate phototransistor memory with perovskite nanocrystals embedded in fluorinated polyamic acids†

**DOI:** 10.1039/d4na00939h

**Published:** 2025-02-17

**Authors:** Wei-En Wu, You-Wei Cao, Yu-Chih Hsu, Yan-Cheng Lin, Yang-Yen Yu

**Affiliations:** a Department of Materials Engineering, Ming Chi University of Technology New Taipei City 24301 Taiwan yyyu@mail.mcut.edu.tw; b Department of Chemical and Materials Engineering, Chang Gung University Taoyuan City 33302 Taiwan; c Somapex Biotech. Co. Ltd Kaohsiung Taiwan; d Department of Chemical Engineering, National Cheng Kung University Tainan 70101 Taiwan ycl@gs.ncku.edu.tw; e Advanced Research Center of Green Materials Science and Technology, National Taiwan University Taipei 10617 Taiwan

## Abstract

This study aims to develop a hybrid material using fluorine-containing polyamic acid (PAA) polymers and a perovskite (PVSK) for application in transistor-based photomemory devices to enhance both structural and electrical performance. Adding fluorides to the PAA material creates a structure with Lewis acid–base interactions, improving the interface between PVSK and PAA, reducing defect density in the floating gate dielectric layer, and passivating grain defects. Furthermore, the hydrophobic PAA structure provides an improved crystalline nucleation interface for the semiconductor pentacene, thereby significantly enhancing the hole mobility of the transistor. In electrical performance tests, devices utilizing ODA–6FDA (poly(4,4′-diaminodiphenyl ether-*alt*-4,4′-(hexafluoroisopropylidene)diphthalic anhydride)) as the floating gate exhibited a superior ON/OFF current ratio, approaching 10^6^, compared to other PAA materials, and demonstrated stable dynamic switching currents. Additionally, incorporating fluorides into the PVSK material resulted in a more stable memory window, enabling the devices to maintain excellent performance during cyclic operation and long-term storage stability tests. These findings highlight the potential of combining fluorinated polymers with PVSK materials, further advancing the development and application of optoelectronic materials.

## Abstract 2

This study develops a fluorinated PAA-perovskite hybrid for transistor-based photomemory, enhancing structural and electrical performance.

## Graphical abstract

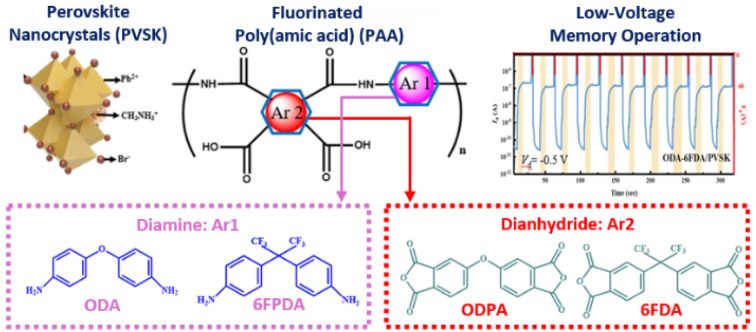

## 1 Introduction

Field-effect transistors (FETs) are fundamental components of modern electronic devices, consisting of three electrodes: the source, drain, and gate. A semiconductor thin film is used as the channel material, with the electric field effect controlling current. These three electrodes work together, and the current channel's formation relies on the electric field's control. Memory devices based on FETs are particularly notable for their fast switching, high current contrast, and operational stability. FET memory devices that use polymers as electrets exhibit good charge-trapping capabilities. However, the real breakthrough comes from the careful selection of materials and the innovative design of the memory devices. Polymer-based memory and organic field-effect transistors (OFET) have potential in wearable electronics, though their performance is insufficient compared with current Si-based devices. The concept of polymer-based memory utilizes a charge-trapping mechanism in OFET structure, similar to charge-trap flash memory. The interaction between additives and polymers in material selection further enhances the charge-trapping efficiency and the size of the memory window in OFETs.^1^ Additionally, using organic/inorganic nanocomposite thin films as floating gates in FETs can significantly improve memory performance. The operating mechanism involves storing charge using nanostructured materials and leveraging the characteristics of nanomaterials, such as the dispersion of nanoparticles in the dielectric layer, combined with the transparency of the materials used, as well as the use of P-type and N-type materials, further enhancing memory performance.^2^

Recent advancements in optoelectronic transistor devices have spurred the development of related materials. FETs can acquire photoresponse and memory capabilities by utilizing photosensitive electrets or floating gate materials. The latest progress in phototransistors and their various applications, including non-volatile memory, artificial synapses, and photodetectors, demonstrates that by regulating the volatility of the floating gate dielectric and the hysteresis effect of the semiconductor, these FETs can be widely applied to different types of devices.^3^ To enhance charge capture capability and channel layer compatibility, the design of polymer electrets or floating gate structures, combined with energy level matching and morphology control, can effectively improve photoresponse, leading to photomemory devices with larger memory windows and bistable current states.^4^

Incorporating various photosensitive or photoresponsive materials into the floating gate dielectric within polymer electrets or dielectric layers can create stable supramolecular or floating gate structures, providing consistent charge capture capability.^5^ When combined with different polymers, the charge-trapping layer of synaptic transistors can also exhibit excellent photoresponse and short-term memory performance.^6^ Chen *et al.*^7^ demonstrated the first hybrid composite material composed of perovskite (PVSK) nanocrystals and polystyrene and its application in photomemory. The *in situ* formation of PVSK nanocrystals within the polymer matrix allows them to be uniformly embedded in the polystyrene matrix. This design produces good photoresponse and an ultra-stable memory ratio of 10^4^ for over 120 days. PVSK has also been applied in electrical-bias-modulated floating-gate memory by forming a direct heterojunction with CdS nanoribbons.^8,9^

Subsequently, many materials, such as PVSK or quantum dots, have been derived for floating gate applications in photoresponsive transistors. In relevant studies, Zhang *et al.*^10^ used PbS quantum dots (QDs) to insulate polymethylmethacrylate to capture charge during photoresponse. Moon *et al.*^11^ incorporated CsPbBr_3_ PVSK in the insulating polystyrene, endowing it with photoresponse and memory characteristics. In addition to PVSK nanomaterials, Wang *et al.*^12^ used fluorescent Si QDs and MoS_2_ heterostructures to exhibit significant synaptic functions. Concerning the dimensions of PVSK nanomaterials, Liao *et al.*^13^ employed 2D Cs_2_Pb(SCN)_2_Br_2_ in combination with different polymers to fabricate memory devices. With regard to the effect of functional groups on insulating polymers, Ercan *et al.*^14^ utilized four different polymers and found that as the size of the PVSK nanocrystals decreased, charge transfer increased, resulting in an ON/OFF current ratio of 10^3^ to over 10^5^. Recently, block copolymers were applied to disperse room-temperature phosphorescent 2D PVSK^15^ or 3D PVSK,^16^ which served as a photosensitive floating gate, resulting in an ON/OFF current ratio of 10^4^. The block copolymers can form a self-assembled microstructure to spatially allocate PVSK nanocrystals within the polymer matrix to enhance the device's photoresponse.

Besides the vinyl-type polymers and block copolymers, various condensation-type polymers can be used as the insulating matrix in the floating gate to accommodate the PVSK nanocrystals. For example, Wu *et al.*^17^ applied poly(amic acid) (PAA) as an insulating matrix to disperse the PVSK nanocrystals. By matching the energy levels and disrupting the interaction between dianhydride and diamine materials, the crystallization ability of PVSK was improved. Based on the studies discussed above, it has been found that using functionalized polymers can significantly enhance the crystallization and dispersion of PVSK nanocrystals in the floating gate thin films, improving overall performance. Chou *et al.*^18^ utilized polyimide (PI) with a high dielectric constant to fabricate transistor-type memory devices that maintained stable ON/OFF states for over 10^4^ seconds. Prior research has highlighted the impact of polymer polar functional group structures on transistor memory performance, particularly in their ability to regulate *in situ* PVSK crystallization.

Fluoropolymers possess excellent electrical insulating properties and ambient stability, such as poly(perfluorobutenylvinylether), commercially known as Cytop (Asahi Glass Co.).^19^ It has been demonstrated that the influence of semiconductor—dielectric affinity on the interfacial characteristics, including the crystalline micro/nanostructure of the semiconductor layer, charge modulation, and charge trapping at the interface, significantly affects OFET device performance. The semiconductor–dielectric affinity is controlled by surface hydrophobicity due to dielectric surface functionality.^20^ Therefore, the introduced fluorine atoms in PAAs can effectively modulate their hydrophobicity, surface energy, and energy levels, further influencing the device's stability, interfacial compatibility with the semiconducting channel, and charge-trapping capability in device operations. Additionally, incorporating fluorides has been shown to improve the grain structure of PVSKs.^21,22^ However, to date, no studies have investigated the application of fluorine-containing PAA in PVSK photomemory devices.

In this study, we selected 4,4′-diaminodiphenyl ether (ODA) and 4,4′-(hexafluoroisopropylidene)diamine (6FPDA) as diamine monomers, and 4,4′-oxydiphthalic anhydride (ODPA) and 4,4′-(hexafluoroisopropylidene)diphthalic anhydride (6FDA) as dianhydride monomers, to develop a PVSK nanocomposite transistor system. PAA was used as the insulating polymer matrix, and the PVSK nanocrystals were encapsulated within the floating gate dielectric of the device. The defect density was investigated within these materials by synthesizing four distinct PAA compositions and introducing fluorides, which enhanced interactions between the carboxyl and amide groups and the PVSK, promoting grain formation and surface improvement. Additionally, the interaction between fluorine atoms and Pb^2+^ in the PVSK crystals led to defect passivation, resulting in more stable grains. The advantages of adding fluorine to PAA contribute to improved growth and electrical stability in memory devices.

## 2 Results and discussion

### 2.1 Analysis of material structure and film hydrophilicity

**Fig. 1:**
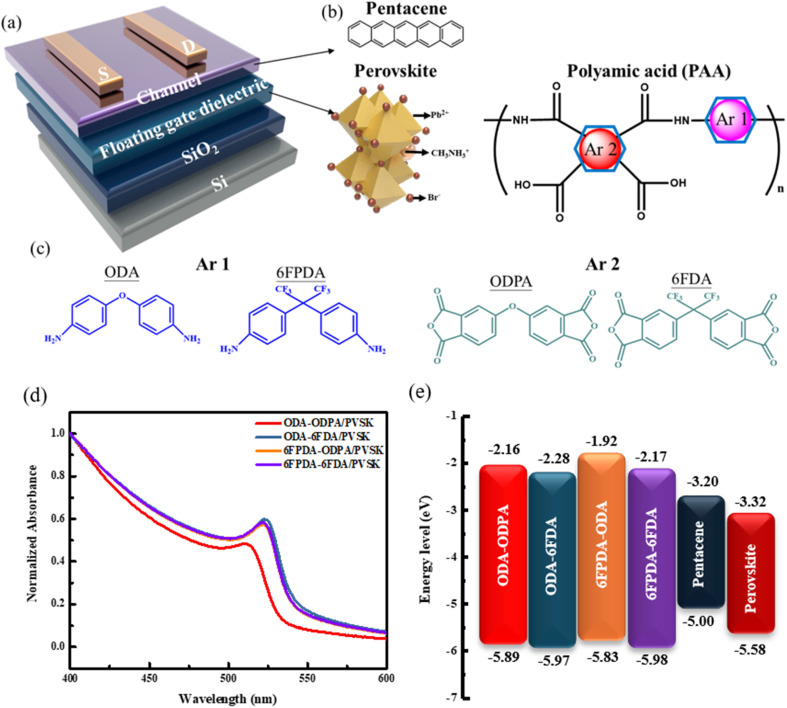
(a) Schematic diagram of the device structure, (b) channel layer and PVSK structure, (c) chemical structures of the diamine and dianhydride monomers in PAA. (d) UV-vis absorption spectrum of PAA/PVSK and (e) energy levels of the constituent materials.

This study chose a 100 nm-thick silicon dioxide layer as the dielectric layer and it was grown on a silicon substrate. The floating gate dielectric was created by mixing four different monomer-structured PAA materials with PVSK (ODA–ODPA/PVSK, ODA–6FDA/PVSK, 6FPDA–ODPA/PVSK, 6FPDA–6FDA/PVSK) using a spin-coating process. The channel layer and electrodes were fabricated using evaporation techniques, with pentacene and gold as the materials. This research explores the impact of fluorine-containing PAA structures on PVSK's grain growth and the device's electrical performance by regulating the materials' monomer structure. The device structure is shown in Fig. 1a, and the chemical structure and composition of the PAA monomer structures are illustrated in Fig. 1b and c.

Contact angle analysis was employed to measure the hydrophilicity and hydrophobicity of the materials, specifically focusing on four different PAA combinations: ODA–ODPA, ODA–6FDA, 6FPDA–ODPA, and 6FPDA–6FDA. DI water and diiodomethane were dropped onto the film surfaces during the experiment, and the resulting contact angles were observed. As shown in Fig. S1,† the 6FPDA–6FDA combination exhibited a water contact angle of 71.9°, indicating that it is more hydrophobic than the other materials. Further examination of the contact angle data (Table S1†) reveals that the 6FPDA–6FDA combination has the lowest surface energy, highlighting a strong correlation between surface energy and hydrophobicity.

Surface energy represents the energy required to overcome molecular forces during material preparation, closely related to hydrophobicity. Hydrophobicity reflects a material's resistance to water adsorption and tendency to avoid contact with water. Generally, the lower the surface energy of a material, especially if it is below the surface tension of water, the more hydrophobic the material is. In contrast, if the material's surface energy is higher, approaching or exceeding the surface tension of water, the material will more readily interact with water, displaying hydrophilicity. According to the data in Table S1,† the excessive addition of fluorine in 6FPDA–6FDA significantly reduces the material's wettability, ultimately achieving the lowest surface energy of 42 mJ m^−2^. In comparison, ODA–6FDA and 6FPDA–ODPA present the second lowest surface energy at 46 and 44 mJ m^−2^, respectively. ODA–ODPA possesses the highest surface energy at 53 mJ m^−2^. It can be clearly seen that the fluorine content directly influences the PAA's surface energy.

### 2.2 Optical analysis of the thin films

The absorption spectra of four PAA/PVSK thin films were analyzed using ultraviolet-visible absorption spectroscopy (UV-vis). After fabrication, the floating gate dielectric and film were spin-coated onto the substrate. According to Fig. 1d, the measurement results indicate that the absorption peaks of the four PAA mixed films are centered around 530 nm, which aligns with previous literature.^23^ Notably, the ODA–ODPA/PVSK material exhibits a pronounced blue shift compared to the other three combinations, suggesting a different absorption peak position for the non-fluorinated combination regarding grain growth. In contrast, the fluorinated combinations show a significant red shift after being incorporated into the PVSK, highlighting a distinct difference in performance.

By combining the UV-vis absorption spectra of the PAA films shown in Fig. S2† with the UPS spectra in Fig. S3,† the optical bandgaps and the highest occupied molecular orbital (HOMO) levels of the PAA films were determined. The HOMO levels for ODA–ODPA, ODA–6FDA, 6FPDA–ODPA, and 6FPDA–6FDA are −5.89, −5.97, −5.83, and −5.98 eV, respectively, while the optical bandgaps are 3.73, 3.69, 3.91, and 3.81 eV, respectively. The lowest unoccupied molecular orbital (LUMO) levels are −2.16, −2.28, −1.92, and −2.17 eV. These findings are summarized in Fig. 1e. The energy levels play a significant role in influencing the optical properties of the PAA and PVSK blended films, as well as the overall performance of the devices.

**Fig. 2:**
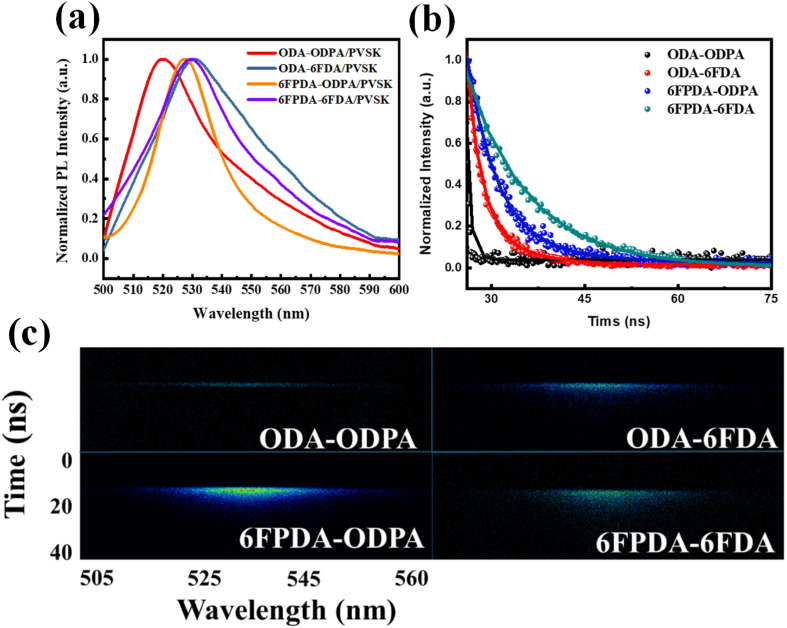
(a) PL emission spectra, (b) 1D TRPL decaying profiles, and (c) 2D TRPL patterns of the PAA/PVSK thin films excited by using a 375 nm laser source.

Next, the four PAA/PVSK composite films were subjected to photoluminescence (PL) testing, as shown in Fig. 2a. PL analysis is closely linked to the previous UV-vis tests, as the excitation source for PL must be selected based on the relevant absorption peaks identified in the UV spectra. Under 375 nm excitation, emission peaks between 500 and 540 nm were observed, with all four composite films showing similar effects. However, a noticeable blue shift was observed in the ODA–ODPA/PVSK combination, suggesting that in the absence of fluorides, the molecular structure of the materials lacks the characteristics imparted by hydrogen bonding and Lewis acid–base interactions. In the 6FPDA–ODPA/PVSK combination, despite having the same fluorine content as ODA–6FDA/PVSK, the results differed, likely due to the presence of fluorine in ODA–6FDA, which promoted the favorable growth of PVSK nanocrystals. Subsequently, Time-Resolved Photoluminescence (TRPL) analysis was conducted on the four PAA/PVSK composite films, as shown in the 2D spectra in Fig. 2b. The spectra of the four combinations reveal significant changes in material lifetimes, progressing from the fluorine-free ODA–ODPA combination to the ODA–6FDA and 6FPDA–ODPA combinations, each containing one fluorinated dianhydride/diamine, and finally to the fully fluorinated 6FPDA–6FDA combination. As shown in the 1D integrated spectra in Fig. 2c, these results, together with an effective charge transfer mechanism, indicate that during the transfer process, more holes are captured from the PVSK into the pentacene within the material's functional groups, thereby reducing the recombination probability and enhancing the photomemory device's sensitivity to light. This demonstrates that adding fluorides has a notable impact on the growth and lifetime of PVSK nanocrystals.

### 2.3 Thin film morphology analysis

**Fig. 3:**
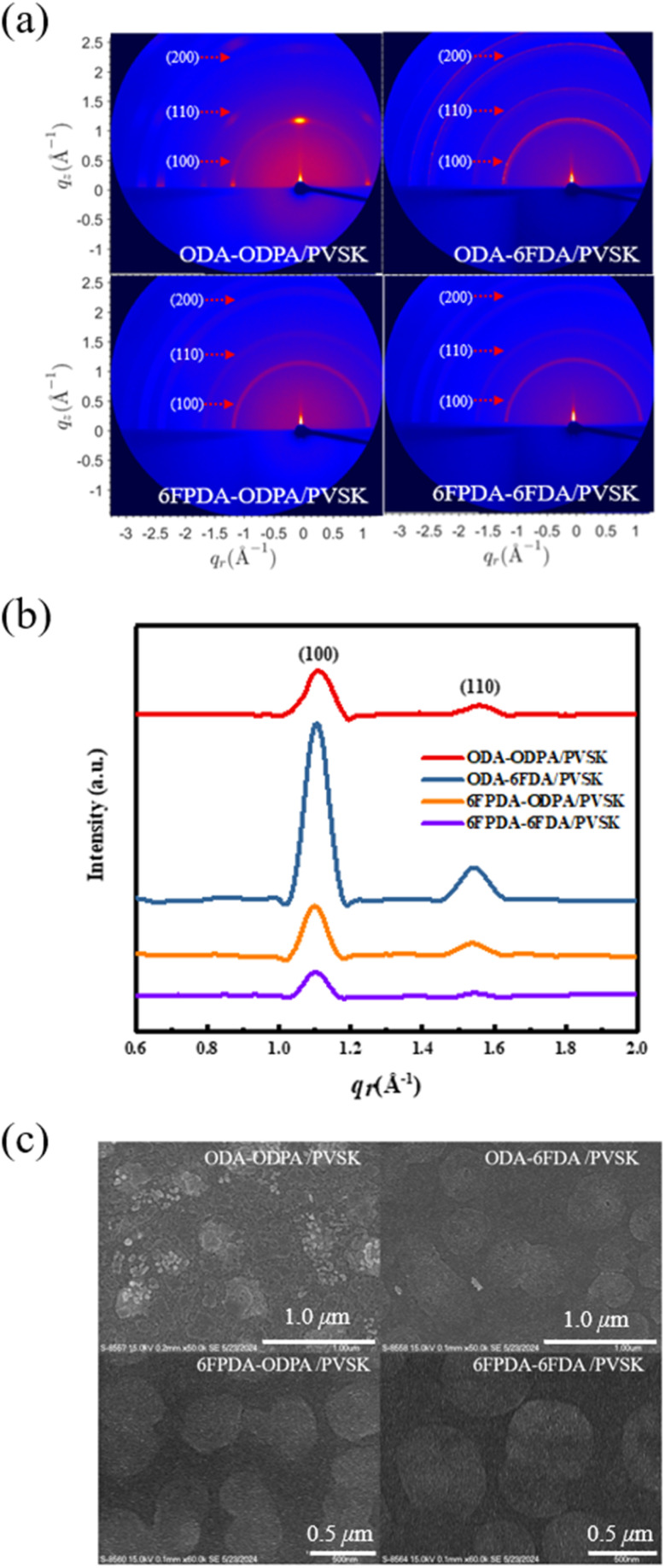
(a) 2D GIWAXS images, (b) normalized 1D GIWAXS full-integration profiles, and (c) FE-SEM images of the PAA/PVSK thin films.

Next, grazing-incidence wide-angle X-ray scattering (GIWAXS) was utilized to analyze the crystal structure of PVSK mixed with fluorinated PAAs, as shown in Fig. 3a. The 1D integrating profiles in Fig. 3b were normalized by the film thickness and exposure time. The diffraction patterns of the four mixed-material films reveal three characteristic peaks at *q* = 1.08°, 1.53°, and 2.16°, corresponding to the (100), (110), and (200) planes, respectively. This experiment confirms that adding fluorine facilitates the growth of PVSK grains within the PAA materials. The ODA–ODPA/PVSK combination exhibited the most distinct characteristic peaks among the materials. However, the direction of polycrystalline grain growth is not the primary focus to improve the performance in floating gate dielectrics. In contrast, fluorine's influence on ODA–6FDA/PVSK is more significant in enriching and producing more stable and uniform grain growth than ODA–ODPA/PVSK. This stability is likely due to the interaction between Pb^2+^ in the PVSK and the added fluorine atoms, which promotes grain growth and defect passivation. Excessive addition of fluorine can disrupt the original grain growth, leading to reduced grain size, decreased crystallinity between grains, and increased lattice defects. These defects, caused by excessive fluorine, may result in structural distortion and significantly reduce subsequent charge transport capabilities. In Fig. 3b, the Bragg law was used to convert the positions of the characteristic peaks in the 2D diffraction patterns into 1D plots of lattice spacings, yielding consistent results. Among the four material combinations, the ODA–6FDA combination, including one fluorinated dianhydride–diamine material, exhibited the highest peak value at the (100) plane in the 1D profiles and 2D patterns. This result indicates that the addition of fluorine significantly impacts PVSK grain growth. Subsequently, the grain size (*L*_c_) of PVSK nanocrystals was estimated using the Scherrer equation. The *L*_c_ values for the four PAA mixed films were determined to be 68.18, 71.54, 73.4, and 75.36 Å, respectively, based on the 1D plots. These results suggest that adding fluorine also influences the growth of PVSK grains in their powder form. Finally, field emission scanning electron microscopy (FE-SEM) was used to observe the surface morphology of the polymer and PVSK mixed films, as shown in Fig. 3c. Among the four combinations, the ODA–ODPA/PVSK film, which lacks fluorine, showed unstable grain growth. In contrast, the other three fluorinated combinations exhibited more stable PVSK grain growth. This observation aligns with the GIWAXS results, indicating that increasing the grain size of the PVSK material can enhance its charge transport performance.

### 2.4 Device characterization of the phototransistor memory

**Fig. 4:**
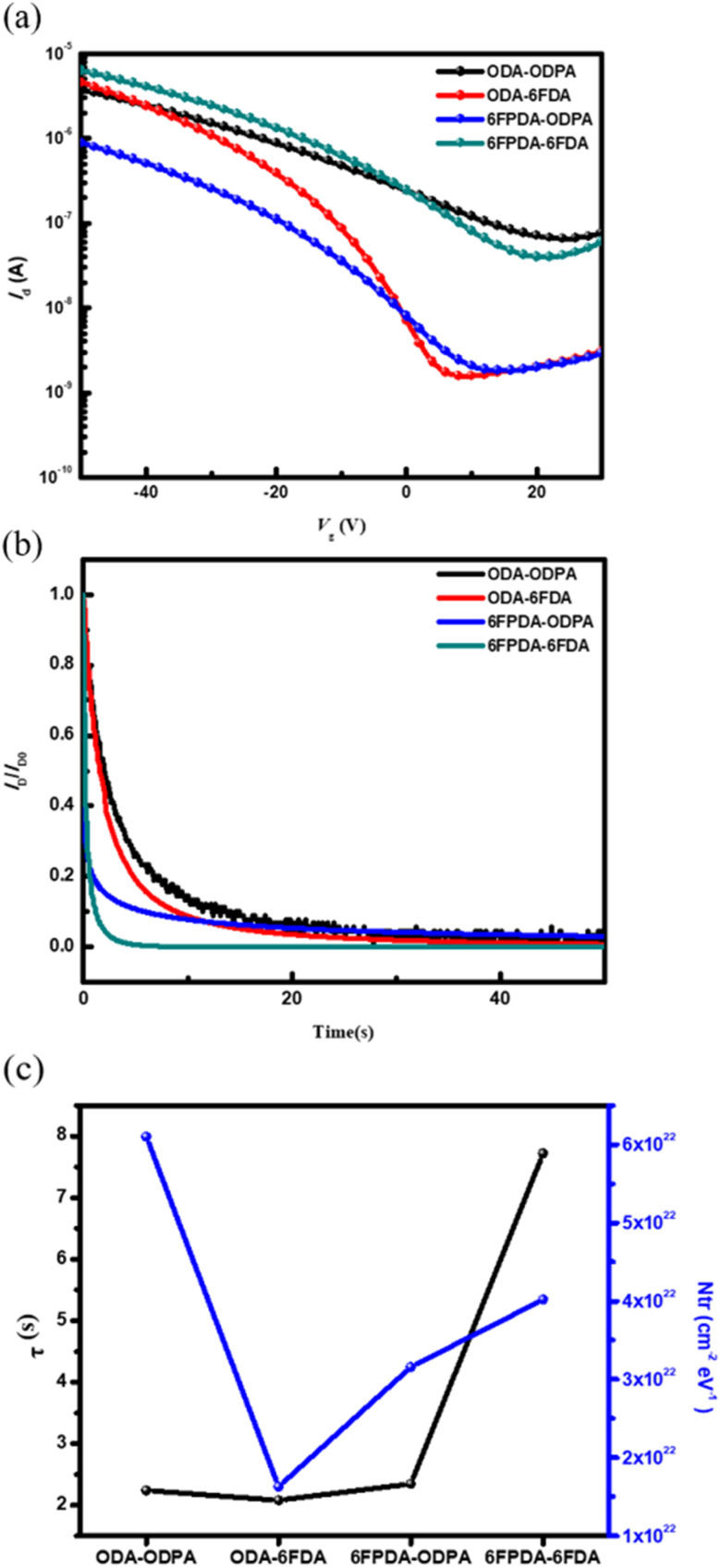
(a) Transfer curves, (b) bias stress stability test at *V*_D_ and *V*_G_ = −10 V, and (c) the characteristic time constant (*τ*) in the bias stress response and the interfacial trap density (*N*_tr_) derived from the subthreshold swing (SS) of the transfer curves of the devices comprising pure PAAs/100 nm-thick SiO_2_ dielectrics and a pentacene channel. The transfer curves were scanned at a fixed *V*_D_ = −40 V from *V*_G_ = 30 to −50 V.

Firstly, four types of PAA films were fabricated in the FET device and used as a dielectric layer. The changes in current over time were tracked by applying a weak bias (*V*_G_ = −10 V and *V*_D_ = −10 V). This test aimed to determine whether the required current variation could be achieved in pure materials when writing with a minimal bias. As shown in Fig. 4b, the material needs to exhibit a slight current variation to reduce the impact of material defects. Therefore, good bias stability is represented by the smallest possible current variation in the pure material during the electrical writing process. Next, a quantitative analysis was performed using the stretched-exponential formula: *I*_D_(*t*) = *I*_D_0__ exp[−(*t*/*τ*)^*β*^],^24,25^ where *τ* represents the time constant, and *β* represents the dispersion force constant. The stretched exponential function applies to dispersive processes in which local environments, such as trapping energies, give rise to a distribution of *τ*, with *β* indicating its degree of variation; *β* = 1 describes the exponential function with a singular *τ* and *β* = 2 describes the normal distribution.^26,27^ The obtained data exhibit a clear trend (see Table 1). Based on the results, the performance of the dielectric layer in the device requires the material to have a smaller *β* value and a more considerable *τ* value, indicating that the material has a lower defect density after applying bias. To understand the rate of current change when the transistor switches from the off state to the on state, the subthreshold swing (SS) was referenced from a formula, an essential parameter for evaluating memory device performance. SS is defined as the gate voltage change required to achieve a one-order magnitude change in current, with the calculation formula: SS = d*V*_G_/(d(log *I*_DS_)). By taking the logarithm of the data in Fig. 4a and fitting the curve to the voltage region, the SS value was estimated, allowing further analysis. Assuming that the interface trap density is independent of energy, the maximum density of interface traps (*N*_tr_) can be calculated using the following formula: *N*_tr_ = *C*_i_/*q*[*q*SS/*kT* ln(10) − 1].^28,29^ Here, *C*_i_ is the areal capacitance of the SiO_2_ dielectric, *k* is the Boltzmann constant (1.38 × 10^−23^ J K^−1^), *q* is the elementary electron charge (1.602 × 10^−19^ C), and *T* is the absolute temperature (300 K). After calculating the *N*_tr_ values for these devices, they were compared with the data in Table 1 and Fig. 4c. For the device using ODA–6FDA as the dielectric layer, it is inferred that the current change at the semiconductor/dielectric interface when the transistor switches from off to on is smaller compared to that of other materials. It also exhibits a lower *N*_tr_ value. Combined with the contact angle and surface energy performance, the material shows good hydrophobicity, and the *β* and *τ* values are well-coordinated. This indicates that this PAA and semiconductor combination is more favorable for charge transport. This disparity between PAAs can be attributed to the interfacial compatibility with pentacene. It has been reported that the surface energy of pentacene is around 48 mJ m^−2^, which is close to that of ODA–6FDA (46 mJ m^−2^). In comparison, 6FPDA–ODPA has the second closest surface energy of 44 mJ m^−2^. Therefore, these two PAAs show the lowest *N*_tr_ values among the PAAs. The interfacial compatibility influences pentacene's crystalline micro/nanostructure and charge-trapping ability at the interface, significantly affecting the OFET and memory performance.^20^

**Table 1:** The device parameters of the four types of PAA used as dielectric layers and the electrical performance parameters of the FET devices

PAA types	*τ* (s)	*β*	SS (V per decade)	*N*_tr_ (cm^−2^ eV^−1^)
ODA–ODPA	2.24	0.47	40.11	6.10 × 10^22^
ODA–6FDA	2.08	0.59	10.71	1.63 × 10^22^
6FPDA–ODPA	2.35	0.75	20.13	3.16 × 10^22^
6FPDA–6FDA	7.72	0.55	25.97	4.02× 10^22^

**Fig. 5:**
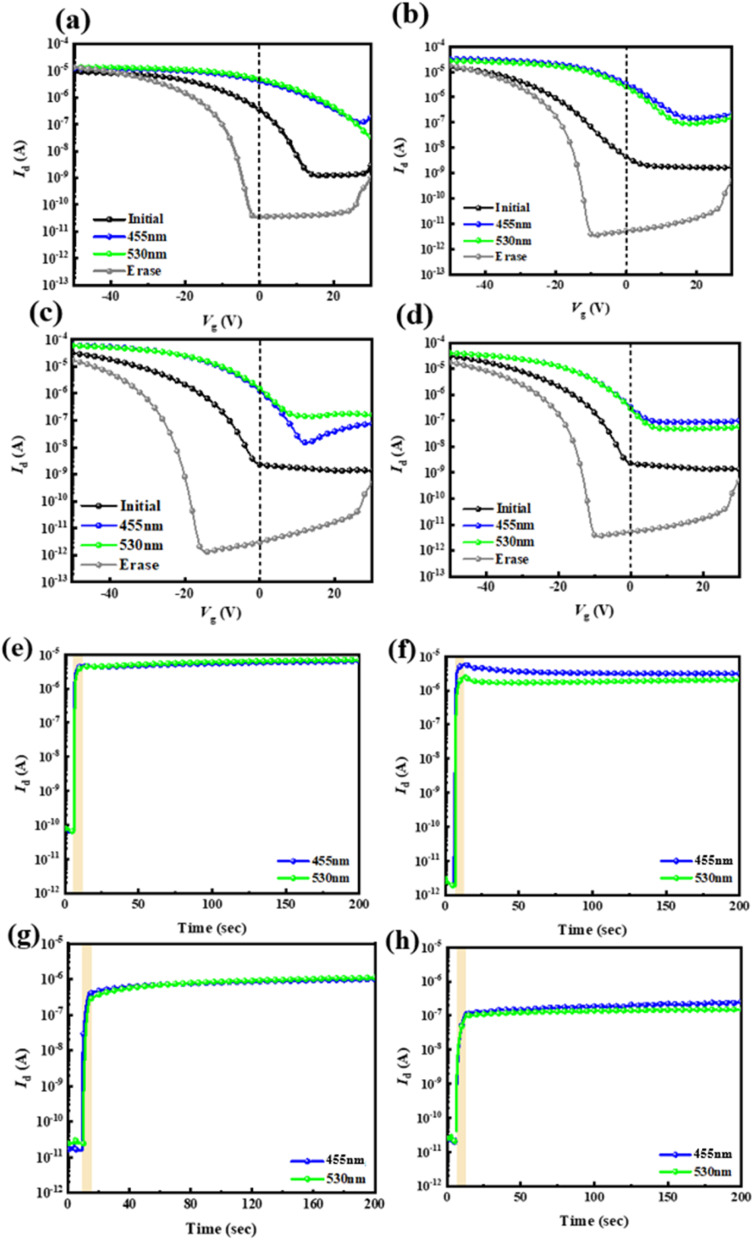
(a–d) Transfer characteristics and (e–h) temporal current characteristics hybrid floating-gate photomemory devices using four types of PAA/PVSK thin films as the floating gate: (a and e) ODA–ODPA, (b and f) ODA–6FDA, (c and g) 6FPDA–ODPA, and (d and h) 6FPDA–6FDA. The photowriting was conducted at a fixed *V*_D_ = −40 V by applying 455 nm (12.5 mW cm^−2^) or 530 nm (16.0 mW cm^−2^) light for 10 s. The electrical erasing process used a *V*_G_ pulse of −60 V at *V*_D_ = 0 V for 1 s.

Next, the four types of thin hybrid films (PAA/PVSK) were applied as floating gate dielectrics for the phototransistor memory devices. These phototransistor memory devices can trap charges by leveraging the exciton generation and charge dissociation behavior of PVSK grains combined with the photoresponsive properties of the floating gate. The transfer curves and transient curves of the memory devices are shown in Fig. 5a–d and e–h, respectively. The material properties were observed by driving the electrodes at an initial state of *V*_D_ = −50 V and applying a bias ranging from *V*_D_ = 30 to −50 V to observe the changes in electrode current. The black line in the figures represents the initial state, the blue line represents writing with a blue light source, and the green line represents writing with a green light source, with the rectangular block indicating the illuminated region. The gray line represents the device being electrically erased under *V*_G_ = −60 V and *V*_D_ = 0 V for 1 second. Before optical writing, electrical erasure with *V*_G_ = −60 V is required, followed by a 10 second wait to stabilize the dark current and then 10 seconds of photowriting. The threshold voltages (*V*_th_) after electrical erasing (*V*_th,erase_) at *V*_G_ = −60 V for 1 s or photowriting (*V*_th,write_) with light illumination for 10 s at *V*_D_ = −40 V, and the corresponding memory window, Δ*V*_th_ = *V*_th,write_ − *V*_th,erase_, were calculated and are presented in Table 2. Observing these electrical properties, the carrier mobility (*μ*_h_) and *V*_th_ were calculated based on the relationship between IDS and *V*_G_ in the saturated state, providing relevant data for the four PAA/PVSK hybrid floating-gate memory devices.

**Table 2:** Device parameters of the phototransistor memory comprising the PAA/PVSK floating gate dielectric and a pentacene channel layer

	Light wavelength (nm)	ODA–ODPA/PVSK	ODA–6FDA/PVSK	6FPDA–ODPA/PVSK	6FPDA–6FDA/PVSK
*μ*_h_ a (cm^2^ V^−1^ s^−1^)	—	0.016	0.031	0.060	0.056
*V*_th,erase_ b (V)	—	−3.58	−14.71	−22.09	−14.39
*V*_th,write_ b (V)	455	29.93	14.26	7.10	7.34
	530	31.14	12.50	8.10	6.44
Δ*V*_th_^c^ (V)	455	33.51	28.97	29.19	21.73
	530	34.72	27.21	30.19	20.83
*I*_ON_/*I*_OFF_^d^	455	3.56 × 10^4^	1.05 × 10^6^	1.47 × 10^4^	4.36 × 10^3^
	530	2.92 × 10^4^	7.84× 10^5^	8.96 × 10^3^	3.13 × 10^3^
*R* e (A W^−1^)	455	0.76	0.99	0.07	0.02
	530	0.70	0.45	0.04	0.02
*S* f	455	7.2 × 10^4^	3.2 × 10^6^	2.7 × 10^4^	6.8 × 10^3^
	530	6.6 × 10^4^	1.5 × 10^6^	1.2 × 10^4^	5.8 × 10^3^
EQE^g^ (%)	455	18.6	270.8	216.7	5.5
	530	9.4	136.3	136.1	4.7

a Mobility derived from the transfer characteristics' saturation regime in the initial state.

b Threshold voltage after electrical erasing (*V*_th,erase_) at *V*_G_ = −60 V for 1 s or photowriting (*V*_th,write_) with light illumination for 10 s at *V*_D_ = −40 V.

c Δ*V*_th_ = *V*_th,write_ − *V*_th,erase_.

d Current contrast defined at *V*_G_ = 0 V of the transfer characteristics after electrical erasing or photowriting.

e *R* = (*I*_light_ − *I*_dark_)/(*P*_in_*A*), where *I*_light_ and *I*_dark_ are the currents before and after photowriting, *P*_in_ is the light intensity, and *A* is the channel area.

f *S* = (*I*_light_ − *I*_dark_)/*I*_dark_.

g EQE = (*R* × *h* × *c*)/(*e* × *λ*), where *h* is the Planck constant, *c* is the speed of light, *e* is the elementary charge, and *λ* is the light wavelength.

The PAA/PVSK material absorbs light in the floating gate dielectric to generate excitons, dissociating into electrons and holes. Since the channel material pentacene is P-type, it attracts the holes generated in the floating gate dielectric while the electrons remain in their original positions. The transient curves allow observation of the device's response after illumination. It is worth noting that the PVSK-based floating gate dielectric is prone to forming a built-in electric field that shifts the transfer curves to the positive region. The drain current increment without light illumination is associated with the shifted transfer curves to the positive region. Some factors contribute to this phenomenon: (i) electric-field induced Joule heating, (ii) pinhole formation under electrical bias, and (iii) halogen ion migration toward the interface. Considering the low vertical bias applied to the floating gate dielectric, this behavior is primarily attributed to the ion migration induced by the electrical stress from the source/drain voltage, which outputs the hole carriers from the floating gate to the channel. The HOMO gaps between PAAs and the perovskite are relatively narrow; therefore, the ion migration in the floating gate dielectric will easily induce electron traps.^30,31^ This is why there is an abnormal increment in the transient characteristics' drain current after light illumination. However, structure designs can mitigate this propensity, and ODA–6FDA performs at the highest stability among the PAAs due to the higher-lying LUMO level and lower-lying HOMO level than other PAA analogs.

The parameters of *R* (responsivity), *S* (sensitivity), and EQE values (external quantum efficiency) provide significant advantages in memory devices, particularly in optoelectronic memory applications. High *R* values are observed to enhance the ability of memory devices to detect and amplify weak optical signals, which is crucial for efficient light-induced programming and reading processes. A higher *S* value improves the device's signal-to-noise ratio, enabling more precise data storage and retrieval even under low-intensity illumination or high background noise conditions. Furthermore, a high EQE value reflects efficient photon-to-electron conversion, thereby enhancing the memory device's overall energy efficiency and responsiveness during optical operations. These advantages have been demonstrated in previous studies, such as those investigating photodetectors and phototransistors, where the significance of these parameters in improving device performance has been extensively discussed.^32,33^ Among the four PAA/PVSK floating-gate devices, the ODA–ODPA floating-gate device exhibits poor charge transport ability due to the lack of fluorine in its two PAA monomer materials, preventing interaction with Pb^2+^ during grain growth. In contrast, the ODA–6FDA floating-gate device shows the best optical memory effect. This is because adding fluorides allows fluorine atoms in the fluorinated PAA to interact with Pb^2+^ in the PVSK, promoting the growth and crystallization of PVSK nanocrystals. However, excessive fluoride increases defect density in the film, reducing the crystallinity of the grains. The test results show that the ODA–6FDA floating-gate device has the lowest OFF current and the highest ON current, with the best memory ratio (*I*_ON_/*I*_OFF_ = 1.05 × 10^6^), light responsivity (*R* = 0.99 A W^−1^), photosensitivity (*S* = 3.2 × 10^6^), and external quantum efficiency (EQE = 270.8%), attributed to the appropriate fluoride addition. Note that *I*_ON_/*I*_OFF_ is the current contrast defined at *V*_G_ = 0 V of the transfer characteristics after electrical erasing or photowriting; *R* = (*I*_light_ − *I*_dark_)/(*P*_in_*A*), where *I*_light_ and *I*_dark_ are the currents before and after photowriting, *P*_in_ is the intensity of 455 nm (12.5 mW cm^−2^) or 530 nm (16.0 mW cm^−2^) light, and *A* is the channel area of 5 × 10^−4^ cm^2^; *S* = (*I*_light_ − *I*_dark_)/*I*_dark_; EQE = (*R* × *h* × *c*)/(*e* × *λ*), where *h* is the Planck constant of 6.63 × 10^−34^ m^2^ kg s^−1^, *c* is the speed of light of 3 × 10^8^ m s^−1^, *e* is the elementary charge of 1.6 × 10^−19^ C, and *λ* represents the light wavelengths of 455 and 530 nm.^33,34^ In the ON/OFF states, the PAA/PVSK hybrid floating-gate devices synthesized with only one fluorinated diamine or dianhydride monomer showed better ON/OFF current ratios, with the ODA–6FDA/PVSK floating-gate device achieving the highest *I*_ON_/*I*_OFF_. In contrast, the 6FPDA–6FDA/PVSK floating-gate device had a relatively minor memory window. Excessive fluoride reduced the device's voltage tunability, indicating that fluoride significantly impacts the electrical performance of phototransistor memory devices, as can be seen in Table 2.

**Fig. 6:**
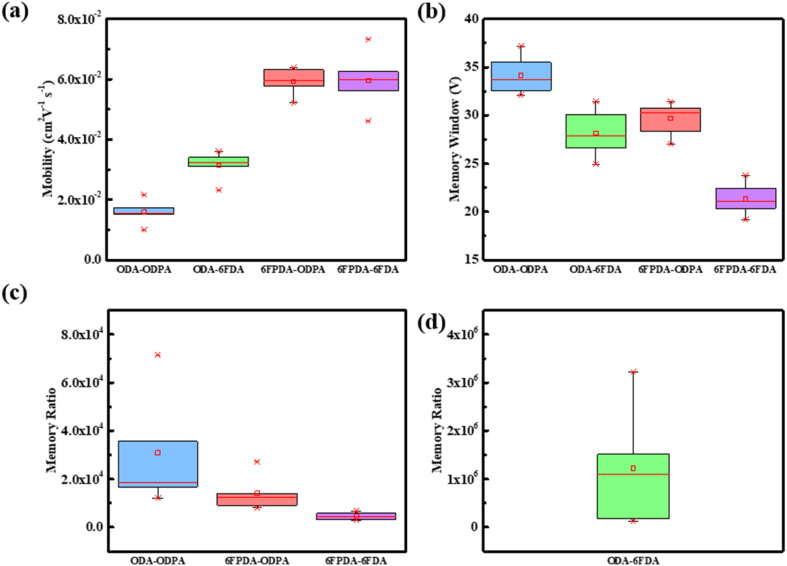
Standard deviation of various electrical parameters for every 5 photovoltaic elements: (a) mobility, (b) memory window, (c and d) memory ratio.

Next, the optoelectronic devices prepared by the four blend films of ODA–ODPA/PVSK, ODA–6FDA/PVSK, 6FPDA–ODPA/PVSK, and 6FPDA–6FDA/PVSK were analyzed. As shown in Fig. 6, for each of the five devices its average standard deviation was calculated, followed by its carrier mobility, memory window, and memory ratio. ODA–ODPA/PVSK has the highest memory window among the optoelectronic devices, with the median falling at 33 V, which means that this combination has the best ability to control the voltage range, while ODA–6FDA has the best high-ON/OFF ratio among the memory ratios. Reaching 10^6^ means that the device can store more charges after being illuminated under the same light intensity. Next, the capacitance of the four pure PAA materials was also analyzed. The capacitance of ODA–ODPA was 18.8 nF cm^−2^, the capacitance of ODA–6FDA was 25.6 nF cm^−2^, the capacitance of 6FPDA–ODA was 20 nF cm^−2^, and the capacitance of 6FPDA–6FDA was 21.2 nF cm^−2^.

**Fig. 7:**
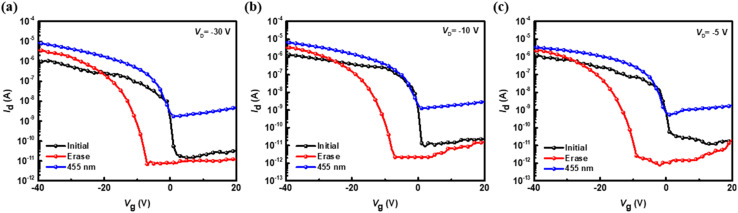
Transfer characteristics of the hybrid floating-gate photomemory device using ODA–6FDA/PVSK as the floating gate under different *V*_D_ for both reading and photowriting: (a) −30 V, (b) −10 V, and (c) −5 V, with an electrical erasing time of 1 s and a photowriting light intensity of 12.5 mW cm^−2^. Note that the photowriting was conducted at *V*_G_ = 0 V.

Next, further tests were conducted on the ODA–6FDA/PVSK device, as shown in Fig. 7, which demonstrate the transfer characteristics (*I*_D_*vs. V*_G_ under varying drain voltage) with *V*_D_ = −30, −10, and −5 V, respectively, for the initial state (black lines), erased state (red lines), and illuminated state at 455 nm (blue lines). At the highest *V*_D_ of −30 V, significant shifts in the transfer curves are observed, with a notable displacement in the *V*_th_ between the initial and erased states and an additional shift caused by illumination. This indicates that more negative *V*_D_ enhances the sensitivity of transfer characteristics, likely due to more pronounced charge trapping and de-trapping effects. At *V*_D_ of −10 V, the shifts in *V*_th_ and the corresponding changes in *I*_D_ between the three states become less pronounced. At *V*_D_ of −5 V, the differences among the initial, erased, and illuminated states are minimal, suggesting a weaker influence of *V*_D_ on charge modulation and transfer characteristics. These results highlight the dependence of transfer curve shifts and drain current magnitudes on *V*_D_ with higher *V*_D_ amplifying the observed differences.

**Fig. 8:**
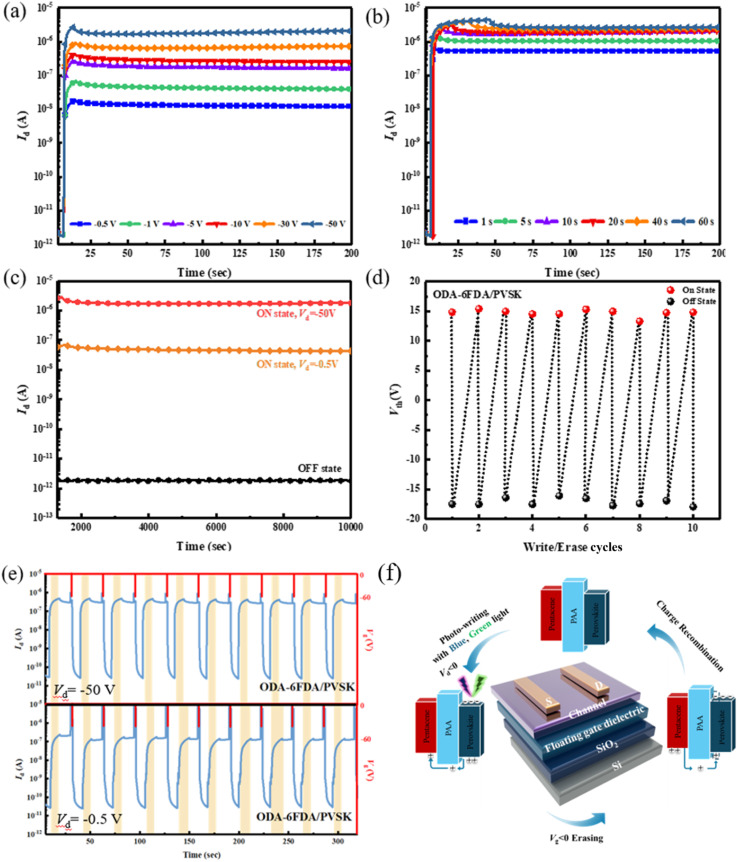
Photoresponse of the ODA–6FDA/PVSK-based device under (a) different *V*_D_ drives with a fixed light exposure time of 10 s and (b) different light exposure times with a fixed *V*_D_ of −50 V. (c) Long-term stability measurement of the device driven by high (−50 V) and low *V*_D_ (−0.5 V) at the ON and OFF states. (d) Threshold voltage transitions and (e) drain current variations along multiple WRER cycles. Note that 455 nm light (12.5 mW cm^−2^) was applied to photowrite the memory device and the electrical erasing process was conducted by applying a *V*_G_ pulse of −60 V at *V*_D_ = 0 V for 1 s. (f) The operational mechanism of the transistor-type photomemory with the PAA/PVSK floating gate dielectric.

Next, further tests were conducted on the ODA–6FDA/PVSK device, as shown in Fig. 8a. The device was driven under different *V*_D_ biases to observe the response and explore the performance differences. Under various *V*_D_ biases, including −0.5, −1, −5, −10, −30, and −50 V, the 455 nm light source was applied for 10 seconds to control the photowriting. The results showed that different biases could all induce a photoresponse, with just −0.5 V, a low voltage sufficient to drive the device and achieve an electrical storage effect, showing an ON/OFF current ratio greater than 10^4^. This indicates that a suitable amount of fluoride addition can enhance the photoresponse of PVSK in memory devices. Finally, device driving for different time durations, with a fixed *V*_D_ = −50 V, was analyzed to test the memory effect. As shown in Fig. 8b, the device could demonstrate an electrical storage effect with just 1 second of writing. These two experimental measurements indicate that the device preparation allows for more fine-tuning possibilities.

### 2.5 Stability analysis of phototransistor memory devices

According to the long-term current stability measurements shown in Fig. 8c, although the method is similar to the transient curve, there are notable differences. After optical writing and electrical erasing, the original test duration of 200 seconds was extended to 10^4^ seconds to observe the stability of the ON and OFF states over a more extended period. The results indicate that even after 10^4^ seconds, the ON and OFF states of the device remain very stable. When driven by either high or low bias, the electrical performance following optical writing shows current stability for more than 10^4^ seconds. This suggests that the device has a strong capacity for long-term charge retention, which is critical for assessing the key performance metrics of optical memory devices. Adding an appropriate amount of fluoride to the material facilitates interaction with Pb^2+^ in the PVSK through Lewis acid–base reactions, which reduces surface defect density, enhances the crystallization of PVSK nanocrystals, and improves the optical response of the PVSK, thereby enhancing device performance.

Subsequently, the device configuration that exhibited the best electrical performance was used to investigate the switching stability of the threshold voltage (*V*_th_) in the optical memory further. Repeated writing and erasing tests were conducted, as shown in Fig. 8d, with ten measurement cycles to assess the memory window's stability. The measurement method was consistent with that used for transient curves. Through WRER (write–read–erase–read) tests of current, long-term current stability, and storage stability, the electrical performance of the device using ODA–6FDA/PVSK as the floating-gate structure was compared under low and high voltage conditions. A WRER test was conducted to evaluate the memory device's retention and permanence. A 455 nm light source was used for 10 seconds of writing, followed by electrical erasure, constituting a complete cycle. To understand the device's switching stability under continuous, identical operation, the measurement results showed that, regardless of the combination of fluorinated diamines or dianhydrides, the device maintained a stable *I*_ON_/*I*_OFF_ after repeated cycles of optical writing and erasing. Conversely, repeated testing revealed an issue with insufficient ON current in PAA materials with either excessive fluorine or no fluorine. This is consistent with previous single write/erase measurement results, suggesting that the differences are due to variations in the crystallization of PVSK grains. PAA materials without added fluoride cannot interact effectively with Pb^2+^ in the PVSK, while excessive fluoride increases film defect density, reduces PVSK crystallization, and degrades the device performance. As shown in Fig. 8e, whether under moderately negative bias (*V*_D_ = −0.5 V) or highly negative bias (*V*_D_ = −50 V), the prepared devices exhibited stable *I*_ON_/*I*_OFF_ of approximately 10^3^ and 10^4^, respectively. This indicates that the device possesses good cycling durability, even under low voltage conditions. Based on the WRER test results, using ODA–6FDA/PVSK as the floating gate dielectric for the optical memory device demonstrated the best electrical performance and device stability.

Finally, as explained in Fig. 8f, this study elucidates the operational process of the transistor-type memory device. When the device is exposed to a light source (blue or green light) for writing, the PVSK absorbs photons, and the resulting excitons generate electrons and holes. Since the channel material, pentacene, is P-type, it attracts the holes from the floating gate dielectric to the channel layer. The current from the source to the drain represents the electrical writing effect. After writing, when a negative *V*_G_ is applied, the holes in the channel layer are attracted back to the PVSK, which neutralizes the original electrons, completing the electrical erasing effect. Concerning the PAA structure design, utilizing a suitable polymer matrix can mitigate the bias instability of ion migration in perovskites. As seen in the energy level alignment, the LUMO gaps between the PAAs, perovskite, and pentacene are sufficiently large to stabilize the trapped charges. ODA–ODPA and 6FPDA–ODPA show low LUMO levels due to the electron-donating ether group in the ODPA dianhydride. The low LUMO level is unfavorable for stabilizing the trapped electrons after photowriting. With a suitable structure design (ODA–6FDA and 6FPDA–6FDA), the HOMO gaps between PAAs and pentacene can reach around 1 eV to avoid hole back-trapping from the channel, warranting good memory stability. The ODA–6FDA shows a slightly higher HOMO level than 6FPDA–6FDA due to the electron-donating ether group in the ODA diamine. Although 6FPDA–6FDA possesses the lowest HOMO level among the PAAs studied, its relatively high *N*_tr_ precludes memory stability. Collectively, the encapsulation of PVSK in the PAA matrix can mitigate the de-trapping of electrons and hole back-trapping from the channel.

## 3 Conclusion

This study successfully fabricated a FET-based phototransistor memory device using a combination of fluorinated PAA and PVSK nanocrystals as a floating-gate dielectric. The research details how adding fluoride to the PAA material impacts the performance of the PVSK memory device. The introduction of an appropriate amount of fluoride causes it to interact with Pb^2+^ in the PVSK through Lewis acid–base interactions, reducing the defect density in the channel layer and passivating grain defects. This enhancement improves the grains' crystallinity, boosts the PVSK's light-harvesting capability, and enhances electrical efficiency. Additionally, the fluorinated and hydrophobic PAA structure provides a superior nucleation interface for the semiconductor pentacene, increasing hole mobility in the transistor. The device utilizing ODA–6FDA as the floating gate demonstrated a significantly better *I*_ON_/*I*_OFF_, approaching 10^6^, compared to other PAA materials. When driven by a shallow bias, this combination maintained stable current performance close to 10^3^ over ten continuous cycles in WRER testing, showing consistent dynamic switching current performance and long-term electrical retention even after more than 10^4^ seconds.

## Data availability

The data that support the findings of this study are available on request from the corresponding authors.

## Author contributions

W.-E. W. fabricated and characterized the memory devices and wrote the original manuscript. Y.-W. C. characterized the morphological properties of the thin films and conducted the formal analysis. Y.-C. H. conducted data curation. Y.-C. L. contributed to the conceptualization of this study. Y.-Y. Y. supervised this project and edited the original manuscript. The manuscript was written with contributions from all authors, and all authors have approved the final version.

## Conflicts of interest

There are no conflicts to declare among the authors.

## Supplementary Material

NA-OLF-D4NA00939H-s001
