# Supplementary material for: Improving the performance of floating gate phototransistor memory with perovskite nanocrystals embedded in fluorinated polyamic acids
Source: Nanoscale Adv. 2025 Feb 17;7(7):2092–104. doi: 10.1039/d4na00939h (PMC11843254; doi:10.1039/d4na00939h)
Supplement: NA-007-D4NA00939H-s001 [file NA-007-D4NA00939H-s001.pdf]

Supplementary Information (SI) for Nanoscale Advances.
This journal is © The Royal Society of Chemistry 2025

Supporting Information

Improving the Performance of Floating Gate Phototransistor Memory with Perovskite Nanocrystals Embedded in Fluorinated Polyamic Acids

Wei-En Wu[1], *You-Wei Cao*[1], *Yu-Chih Hsu* [3], *Yan-Cheng Lin,*[4,5*] *Yang-Yen Yu*[1,2*]

[1] Department of Materials Engineering, Ming Chi University of Technology, New Taipei City 24301, Taiwan.

[2] Department of Chemical and Materials Engineering, Chang Gung University, Taoyuan City 33302, Taiwan

[3] Somapex Biotech. Co. Ltd.Kaohsiung, Taiwan

[4] Department of Chemical Engineering, National Cheng Kung University, Tainan 70101, Taiwan.

[5] Advanced Research Center of Green Materials Science and Technology, National Taiwan University, Taipei 10617, Taiwan.

[*] Corresponding authors: ycl@gs.ncku.edu.tw (Y.-C. L); yyyu@mail.mcut.edu.tw (Y.-Y. Y)

Experimental Section

Materials.

Methylammonium bromide (CH_3NH_3Br, 98%), lead(II) bromide ($PbBr_2$, 99%, trace metal basis), 4,4'-oxydianiline (ODA, 97%), 4,4'-sulfonyldianiline (SODA, 97%), pyromellitic dianhydride (PMDA, 97%), 1,2,3,4-cyclobutanetetracarboxylic dianhydride (CBDA, ≥94%) and *N*, *N*-dimethylformamide (DMF, anhydrous, 99.8%) were purchased from Sigma-Aldrich (Missouri, USA) or Tokyo Chemical Industry (Tokyo, Japan). Pentacene was ordered from Luminescence Technology Corp (Taiwan). All materials were used as received without further purification.

Device Fabrication.

The preparation steps are as follows: Firstly, the silicon/SiO_2 substrate was thoroughly cleaned by sonicating in DI water, acetone, and isopropanol for 15 minutes each. Subsequently, the cleaned substrate underwent a 10-minute cleaning process in a plasma cleaner (Harrick Plasma, PDC-32G). For the memory layer, four different 15 wt% PAA precursor solutions (ODA-PMDA, ODA-CBDA, SODA-PMDA, SODA-CBDA) were prepared using commercially available ODA, SODA, PMDA, and CBDA monomers in a 1:1 molar ratio for diamine: dianhydride. The mixtures were stirred on a magnetic stirrer for at least 16 hours. Then, a PAA precursor solution with a concentration of 30 mg ml^{-1} and a PAA: $MAPbBr_3$ weight ratio of 1:3 was prepared in DMF solvent and stirred for 30 minutes. Spin coating was performed in three stages: initially, at 0 rpm for 10 seconds to ensure even distribution on the substrate surface, followed by 2000 rpm for 60 seconds, and finally, at 3000 rpm for 10 seconds to dry the excess solution and form a film. The samples were subsequently annealed at 120°C for 10 minutes to promote the growth of perovskite crystals. For the transport layer, pentacene was thermally evaporated onto the substrate under a vacuum pressure of 3×10^{-6} torr. The deposition rate was maintained at 0.2−0.3 Å s^{-1}, resulting in a 50-nm thin film. After masking, gold electrodes with

dimensions of 1 mm in width (*W*), 0.1 mm in length (*L*), and 70 nm in thickness were deposited onto the transport layer at a rate of 0.4−0.5 Å s^{-1}.

Characterization.

The UV–Vis absorption spectra were recorded using a Jasco V-650 UV-Vis spectrophotometer. The emission wavelength of the films was measured using a Fluoromax-4 fluorescence/phosphorescence spectrometer with an excitation wavelength of 365 nm. The functional groups of the films were analyzed using a Spectrum One Fourier Transform Infrared (FTIR) spectrometer. The HOMO levels of the PAAs were determined from CV using a CHI 6273E electrochemical analyzer, and the LUMO levels were calculated from the optical bandgap derived from the absorption onset of UV–vis absorption. The surface morphology of the polymer films was analyzed using an S-5200 high-resolution scanning electron microscope (SEM) and atomic force microscopy (AFM, Bruker Innova) operating in tapping mode. The device performance was measured using a Keithley 2634B instrument controlled by a computer inside a glovebox. The light source for device programming was provided by MIGHTEX. The device current was collected at fixed drain voltage (V_D = −40 V), and photowriting was conducted by applying 455-nm (12.5 mW cm^{-2}) or 530-nm (16.0 mW cm^{-2}) light for 10 s at V_D = −40 V. The intensities of the light sources were calibrated by a laser power meter (Thorlabs PM 100D). The electrical erasing process was conducted by applying a gate voltage (V_G) of −60 V at a V_D of 0 V for 1 s. The transfer curve of the device was derived, and the hole mobility (μ_h) and threshold voltage (V_{th}) were evaluated from the saturation regime according to the following equation: $I_D = \mu_h \frac{W}{2L} C_{ox}(V_G - V_{th})^2$ where the C_{ox} is the areal capacitance of a 100-nm-thick SiO_2 layer (31.5 nF cm^{-2}, *W* is the channel width of 1000 µm, *L* is the channel length of 50 µm.

Table S1. Summary of the contact angles and surface energy of the PAA films.

	θ_{Water}[°]	θ_{DIM}[°]	γ_{Polar}(mJ/m^2)	$\gamma_{Dispersive}$(mJ/m^2)	γ_{Total}(mJ/m^2)
**ODA-ODPA**	59.16	13.46	2.619	49.93	52.55
**ODA-6FDA**	69.08	32.02	2.530	43.62	46.15
**6FPDA-ODPA**	70.59	36.69	2.181	41.66	43.84
**6FPDA-6FDA**	71.90	43.94	3.676	38.33	42

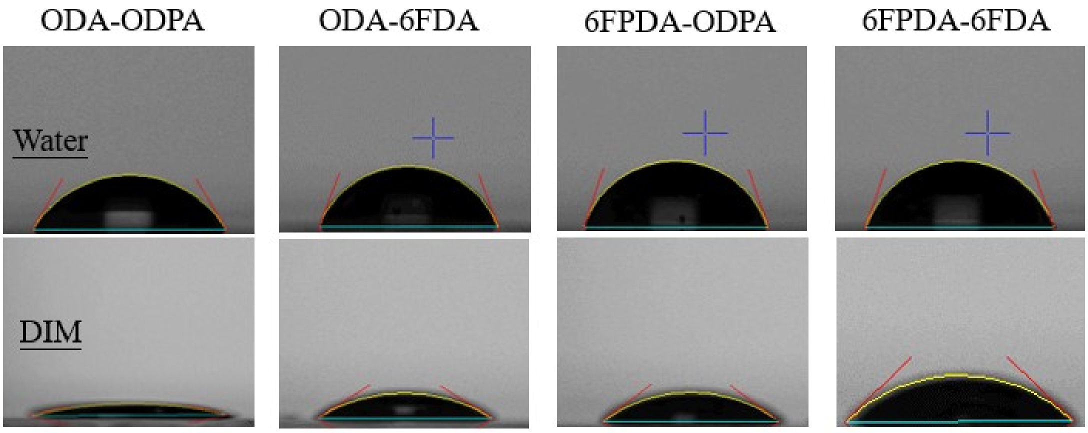

Fig. S1. Contact angle images of a water droplet (top) and a diiodomethane droplet (bottom) on the pure PAA film.

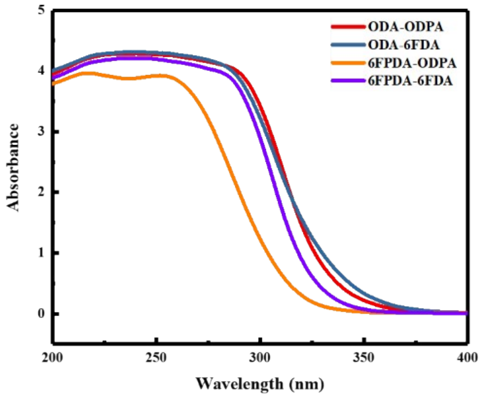

Fig.S2. UV-vis absorption spectrum of the pure PAA films.

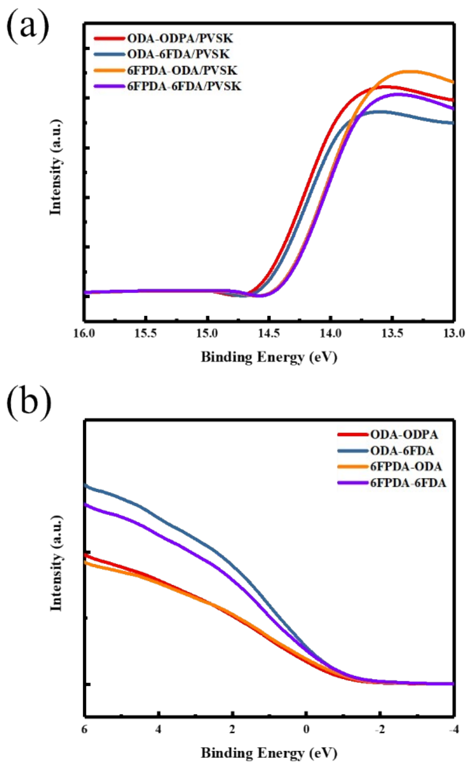

Fig. S3. UPS spectrum of the pure PAA films in the (a) high and (b) low binding energy ranges.
